# Seismogenesis of dual subduction beneath Kanto, central Japan controlled by fluid release

**DOI:** 10.1038/s41598-017-16818-z

**Published:** 2017-12-04

**Authors:** Yingfeng Ji, Shoichi Yoshioka, Vlad C. Manea, Marina Manea

**Affiliations:** 1 Research Center for Urban Safety and Security, Kobe University, Rokkodai-cho 1-1, Nada ward, Kobe, 657-8501 Japan; 2 Department of Planetology, Graduate School of Science, Kobe University, Rokkodai-cho 1-1, Nada ward, Kobe, 657-8501 Japan; 3 Computational Geodynamics Laboratory, Centro de Geociencias, Universidad Nacional Autónoma de México, Campus Juriquilla, Querétaro, 76230 Mexico; 4 Astronomical Institute of the Romanian Academy, 040557 Bucharest, Romania

## Abstract

Dual subduction represents an unusual case of subduction where one oceanic plate subducts on top of another, creating a highly complex tectonic setting. Because of the complex interaction between the two subducted plates, the origin of seismicity in such region is still not fully understood. Here we investigate the thermal structure of dual subduction beneath Kanto, central Japan formed as a consequence of a unique case of triple trench junction. Using high-resolution three-dimensional thermo-mechanical models tailored for the specific dual subduction settings beneath Kanto, we show that, compared with single-plate subduction systems, subduction of double slabs produces a strong variation of mantle flow, thermal and fluid release pattern that strongly controls the regional seismicity distribution. Here the deepening of seismicity in the Pacific slab located under the Philippine Sea slab is explained by delaying at greater depths (~150 km depth) of the eclogitization front in this region. On the other hand, the shallower seismicity observed in the Philippine Sea slab is related to a young and warm plate subduction and probably to the presence of a hot mantle flow traveling underneath the slab and then moving upward on top of the slab.

## Introduction

**Figure 1:**
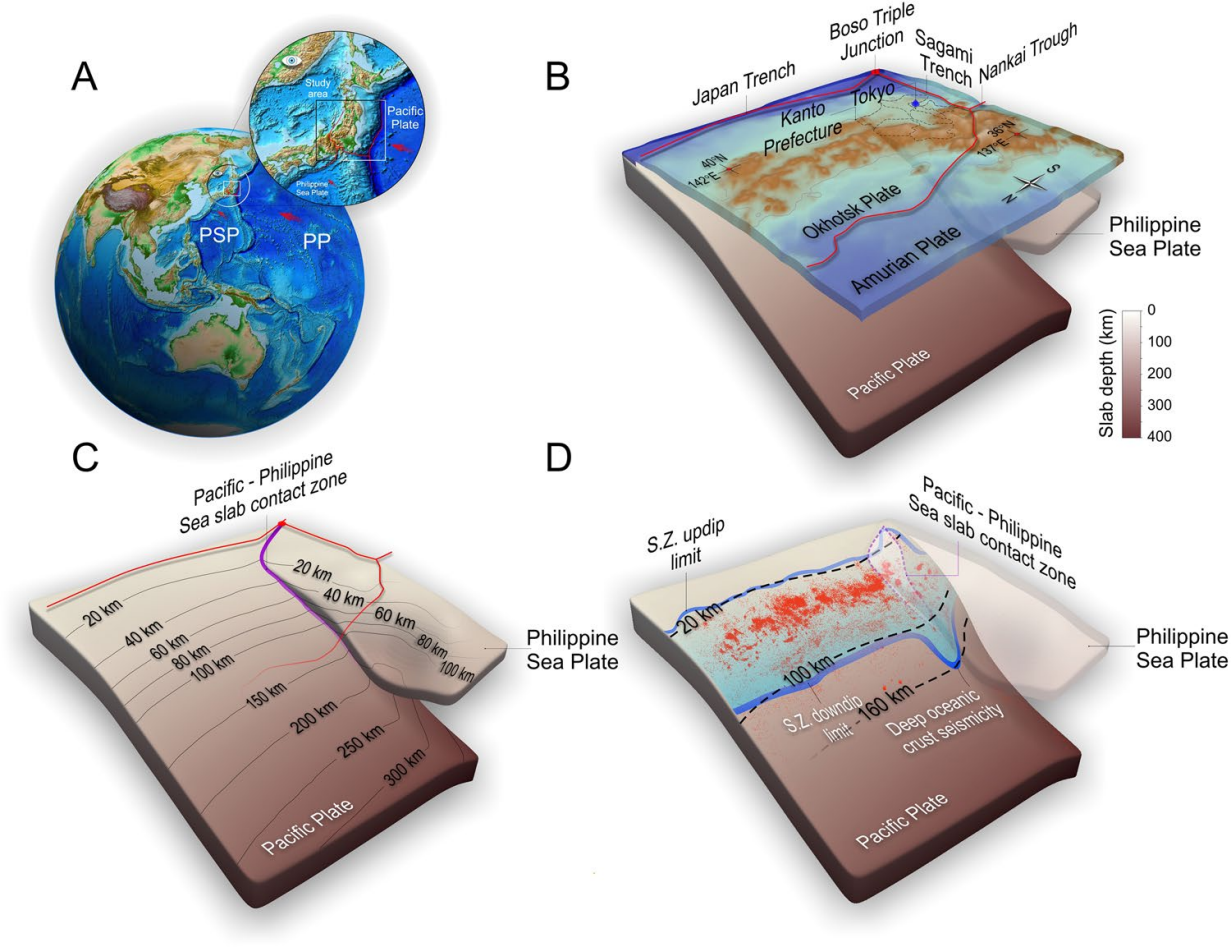
Location of the study area where the Philippine Sea plate subducts on top of the Pacific plate. (**A**) Color-shaded global bathymetry and topography map (ETOPO1 Global Relief Model dataset^45^). The figure was created with the open source software ParaView (http://www.paraview.org) version 5.0.1, licensed under the CC BY 4.0 license (https://creativecommons.org/licenses/by/4.0/). The study area is marked by the white rectangle. (**B**) 3-D view of the study area. Red curves mark the main plate boundary. All 3-D models are visualized from a northwest to southeast viewing angle. (**C**) Geometry of the Pacific and Philippine Sea slabs corresponding to the study area. (**D**) Pacific oceanic crust seismicity (from the unified hypocenter data catalogue (1997.10-2015.2) with magnitude no less than 2.0 from Japan Meteorological Agency) plotted on top of the slab (red dots). The Philippine Sea plate is shown as semitransparent surface. Dark blue rubber bands mark the updip and downdip limit of the crustal seismicity. PP- Pacific Plate, PSP – Philippine Sea Plate.

Dual subduction zones, where two tectonic plates subduct with different rates and azimuths, represent a special case of subduction^1^. This particular case of subduction is formed as a consequence of a triple trench junction, where two oceanic plates simultaneously subduct beneath the overriding plate, and at the same time one beneath the other. Presently, there is only one case of triple trench junction (TTT) known on the Earth, Boso-Oki Triple Junction, located off the coast of Japan beneath the Kanto district^2^. This tectonically complex region is situated where the Philippine Sea plate is subducting beneath the continental North American plate, and at the same time the Pacific plate is subducting below both the Philippine Sea and continental plates along the Japan trench^3^ (Fig. 1B). The interplate seismic activity in this region is characterized by numerous large earthquakes (M > 7), abundant clustered microseismicity, as well as deep-focus intraslab seismicity (~160 km depth) (Fig. 1D), whose origin is still a subject of debate. Comprehensive high-resolution seismic velocity studies obtained based on seismic tomography^4–7^ improved considerably our understanding of seismotectonics in this complex region. The great diversity of seismic activity has been debated in terms of frictional and mechanical interactions along the Philippine Sea-Pacific slab contact zone^8,9^, bending/unbending of local contorted Philippine Sea slab^10^, or net slab pull forces^11^. However, recently the origin of intermediate as well as deep-focus seismicity has been linked with dehydration reactions^12^. Dehydration embrittlement has been proposed as a possible mechanism for decreasing effective normal stress and so triggering intermediate-depth earthquakes^13^. Nevertheless, double-subduction related seismogenesis and its potential relationship with slab dehydration at the intermediate-depth remained unclear mainly due to lack in the high-resolution observations at depths and well-constrained numerical modeling. For the Kanto region, high-resolution seismic tomography has recently provided detailed seismic velocity structure for the incoming plate above a depth of ~100 km beneath the seismically active zone^8^. Additionally, in this area high-resolution hypocenter data (≤5 km in depth) (Fig. 1D) has been collected by the Japan Meteorological Agency in last two decades, offering an exceptional opportunity to investigate potential links between seismogenesis associated with geodynamic processes of dual subduction.

Here, we present three-dimensional robustly-constrained high-resolution thermo-mechanical numerical models to simulate the physical processes associated with simultaneous subduction of the overlapping Philippine Sea and Pacific plates beneath Kanto region (Fig. 1B) (more details can be found in Methods and Supplementary material). Compared with our previous study^14,15^, this is a developed study where we include a thorough study on the effect of Philippine Sea plate thickness on the Pacific plate thermal structure, as well as the toroidal mantle flow around the tip of the Philippine Sea plate. The thickness of the Philippine Sea plate is still controversial, but from the studies including the tension-type seismicity distribution^16,17^, nonlinear travel time tomography^18^ and converted seismic wave^19^, a thickness ranging from 50 km to 60 km is more possible. Our models reveal that the thickness of the Philippine Sea plate and complex interaction with the underneath Pacific subducting plate controls the slab dehydration inside the Pacific slab and generates a complex toroidal mantle flow pattern between the two major tectonic plates that represents the first order cause for seismicity distribution beneath Kanto at depths <160 km.

## Results

### Numerical modelling and temperature distribution

**Figure 2:**
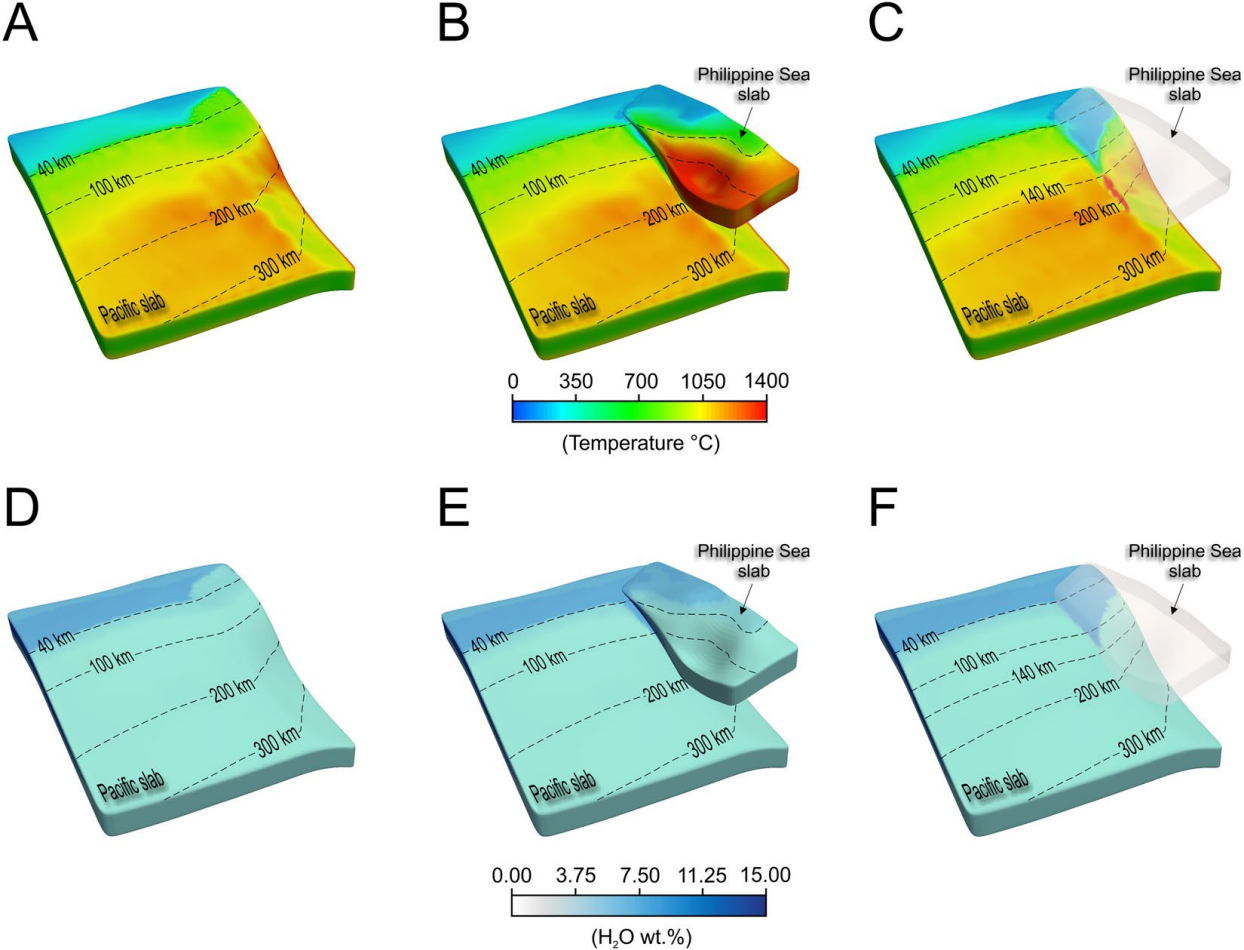
Slab temperature and H_2_O distribution. (**A,D**) Numerical experiment with a single plate (Pacific). (**B,C,E,F**) Numerical experiment with double subduction. In (**C**) and (**F**), the Philippine Sea plate is shown as a semitransparent surface. Note that the water content in (**D**,**E**,**F**) reflects complete slab hydration, whereas in nature slabs are assumed to be partial hydrated^46^. Therefore our estimates should be considered as an upper bound.

To better understand the mantle flow and thermal regimes of the interplate and slab contact zone undergoing subduction upon convergence beneath Kanto region, we developed high-resolution three-dimensional kinematic thermo-mechanical simulations of dual subduction to predict temperature, mantle flow, and spatial distribution of hydrous fluid content inside the Pacific and Philippine Sea subducting plates. The geometry of numerical models is constrained by the present-day dual subducting plate geometry, with specific model parameters illustrated in Supplementary material. Our approach towards evaluating the effect of double subduction involves a set of three-dimensional numerical models including the oblique subduction along a curved oceanic slab specifically chosen for the central Japan^15^. In order to better quantify the effect of double subduction, we also developed a synthetic model where the Philippine Sea plate was intentionally removed from the initial model setup. The calculated thermal structure as well as the fluid content corresponding to both models is presented in Fig. 2. The model without the Philippine Sea plate incorporated shows a thermal distribution of the Pacific plate increasing with depth and running almost parallel to the slab geometry with some perturbations due to the contortion of the Pacific slab at depths >200 km (Fig. 2A). On the other hand, one of the first effects of the Philippine Sea plate subduction on top of the Pacific plate is a significantly deeper than normal cold interplate zone located on the upper surface of the Pacific plate and confined to the contact area between the two plates (Fig. 2B and C). Compared with adjacent areas unaltered by the presence of the Philippine Sea plate, or with the synthetic model without the Philippine Sea plate (Fig. 2A), the slab contact zone beneath Kanto is colder with nearly 300 °C along its southwestern edge. The cooling effect of the Philippine Sea plate sitting on top of the Pacific plate is transferred inside the Pacific slab through the entire oceanic crust but diminishes rapidly in the lithosphere (Fig. S1). Additionally, we observed a local heating effect at the contact zone due to the strong contortion and sagging of the Philippine Sea plate which places in near contact its hot lithosphere with the Pacific slab surface (Fig. 2C). Therefore, the entire oceanic crust of the Pacific slab is strongly affected by dual subduction beneath Kanto region.

### Dual slab dehydration and seismicity distribution

**Figure 3:**
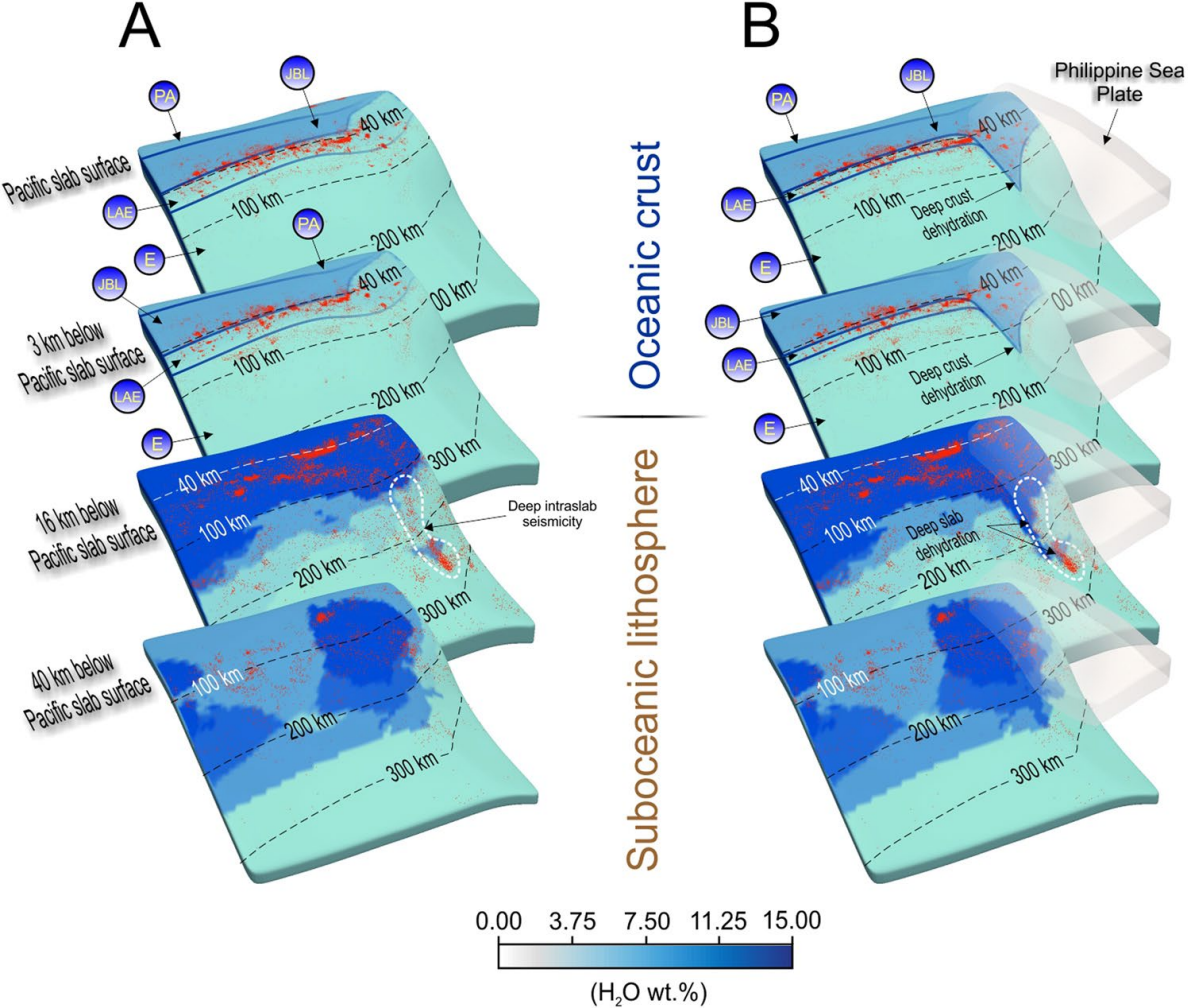
Pacific slab H_2_O and seismicity (red dots) distribution at different slab depth levels. (**A**) Numerical experiment with a single plate (Pacific). Light blue bands mark the metamorphic facies corresponding to the Pacific slab: PA – pumpellyite actinolite, JBL - jadeite blueschist lawsonite, LAE - lawsonite amphibole eclogite, E – eclogite. (**B**) Numerical experiment with double subduction. Philippine Sea plate is shown as a semitransparent surface. White dashed contour defines the location of a deep intraslab earthquake cluster inside the Pacific slab. At each slice the seismicity corresponds to ±2 km measured from the slice surface for the oceanic crust, and $\pm 4\,$ km for the suboceanic lithosphere.

Based on the calculated temperature distribution (Fig. 2A–C), we estimate water content for the Pacific and Philippine Sea slabs, using phase diagrams for MORB and ultramafic rocks such as harzburgite^20–22^. The single subduction model shows a distribution variation of hydrous phases confined above ~40 km depth, and mainly parallel to the Japan Trench (Fig. 2D). To the south, the hydrated portion of the oceanic crust becomes smaller and shallower. Including the subduction of the Philippine Sea plate in the model, the downdip limit of highly hydrous phases inside the Pacific oceanic crust (Fig. 2E and F) increases in the region which corresponds to the double slab contact zone with the Philippine Sea slab beneath the Kanto region. Considering that the temperature drops approximately 300 °C in this region, the presence of stable highly hydrous minerals in the oceanic crust extends to greater depths of ~140 km or more (Fig. 2F). In this depth range, the Pacific plate dehydration and transition to anhydrous eclogite occurs in a relatively short distance. Farther north, both models predict similar results where the oceanic crust transports fluids only to shallower depths of ~40 km, but the water content inside the oceanic lithospheric mantle gradually increases until the Pacific slab reaches ~250 km depth (Fig. 3A and B). Instead, the model with dual subduction indicates that the Philippine Sea plate also strongly controls the water distribution in the oceanic lithosphere in the first ~16 km from the slab surface (Fig. 3B). It is now commonly accepted that the oceanic slabs undergo phase transition and release fluids into the slab contact zone, increasing pore pressure and promote seismogenesis^23,24^. Following, we will investigate the spatial correlation between the slab dehydration and location of seismicity inside the Pacific subducting plate (Fig. 3) using the high accuracy (≤5 km) unified hypocenter data catalogue (1997.10-2015.2) with magnitude no less than 2.0 from Japan Meteorological Agency. One of the first key observations is the presence of a highly active seismic belt located in the oceanic crust and parallel to the Japan trench at a depth of ~40 km. In this region located outside the influence of the Philippine slab, both dehydration models (Fig. 3A and B) show that hydrous minerals are stable and the oceanic crust does not experience any significant phase transformation until it reaches 40 ± 10 km depth (Fig. 3). Within this depth range, the Pacific oceanic crust seismicity correlates remarkably well with our dehydration estimations from jadeite lawsonite blueschist (5.4 wt%), greenschist (3.0 wt%), and lawsonite amphibole eclogite (3.0 wt%) to amphibole eclogite (<1.0 wt%). However, farther south, the synthetic single subduction model that includes only the Pacific plate fails to correlate the main dehydration front location with seismicity distribution in the oceanic crust which actually increases to <150 km depth (Fig. 3A). Instead, the realistic model with dual subduction delays the eclogitization of the crust of the Pacific slab down to ~140 km depth and shows a good correlation between the location of slab dehydration front and deeper (<150 km depth) distribution of seismicity (Fig. 3B). Another interesting aspect of our assessment is the presence of a hydrated strip located inside the Pacific slab, which ranges from ~100 km down to ~250 km more. Figure 3B shows a good correlation between our predicted fluid distribution inside the Pacific slab corresponding to the contact region with the Philippine Sea plate, and the observed intraslab seismicity^25^.

## Discussion

Combining the precise location of earthquakes within the Pacific oceanic crust beneath Kanto region with information of 3-D slab thermal structure and dehydration of double subduction, we found robust evidence of a causal link between oceanic plate dehydration and occurrence of earthquakes (Fig. 3). This is best explained in terms of oceanic crust and lithosphere dehydration beneath Kanto where the slab surface is colder by nearly ~300 °C than that of the model of single subduction (Fig. 2A–C). Compared with the model of single subduction (Fig. 2D), the overlapped double slabs model create a cold slab contact zone, where the phase transition to anhydrous eclogite is shifted to a depth of approximately 140 km (Fig. 2E and F). Wada and He^26^ predicted the same cooling of the PAC-PSP contact zone but at greater depths (70–80 km in Wada and He^26^ vs. 40 km here), which would significantly alter the spatial correspondence between seismicity and slab dehydration. As we will show later, our model setup includes the gap between the Philippine Sea plate and Pacific plate and allows the hot mantle under the Philippine Sea plate to escape under the tip of the slab and creates a toroidal mantle flow.

Dehydration reaction depicted by large seismic velocity changes^21^ accounts for the spatially remarkable seismicity near the slab contact zone, suggesting that eclogite facies phase transformation of hydrous minerals may also facilitate brittle failures, and trigger intense and deeper usual seismic activity beneath Kanto. In order to advance towards a better understanding of seismogenesis, we performed a statistical analysis involving all seismic events recorded by JMA and water content estimates from our numeric simulations. According to this assessment, ~76% of the intra-slab events are accompanied by a slab dehydration rate of >0.02wt%/km and ~62% are associated with a slab hydration of >0.05wt%/km. Additionally for <M3 earthquakes, ~73% are located within regions of slab hydration of >0.05wt%/km. As can be seen from this analysis more than two thirds of the seismic activity is located in regions with a relatively high water content (0.02wt%/km or more), suggesting a strong intrinsic relationship.

Additionally, our modeling results are in good agreement with the location of low S-wave velocity region^8,27^ that can reach a depth range of 120–150 km, which is 80–110 km deeper than the area unaffected by the subduction of the Philippine Sea plate. This depth range is also in good agreement with our estimates of phase transformation of hydrous minerals along the slab contact zone between the Pacific and Philippine Sea plates (Fig. 3B). In fact, our models predict a cooling effect of the Philippine Sea plate that reaches some 16 km inside the Pacific plate (Fig. S1), creating a dehydration strip that extents to a depth of ~250 km. This result explains well the presence of intense Pacific intraslab clustered seismicity in the region that reaches ~280 km depth. It is worth mentioning that our interpretation neglects fluid transport and overlooks any compaction and fluid–solid interaction. Therefore slab dehydration is assumed to induce seismicity in-situ with no migration of fluid. Compaction pressure is an efficient fluid-focusing mechanism and represents a possible mode of focusing and concentrating fluids in subduction zones^28^. Although this might represent a limitation of our modeling results interpretation, they represent a solid base for further testing the ability of compaction pressure to influence fluid migration along slab interface.

**Figure 4:**
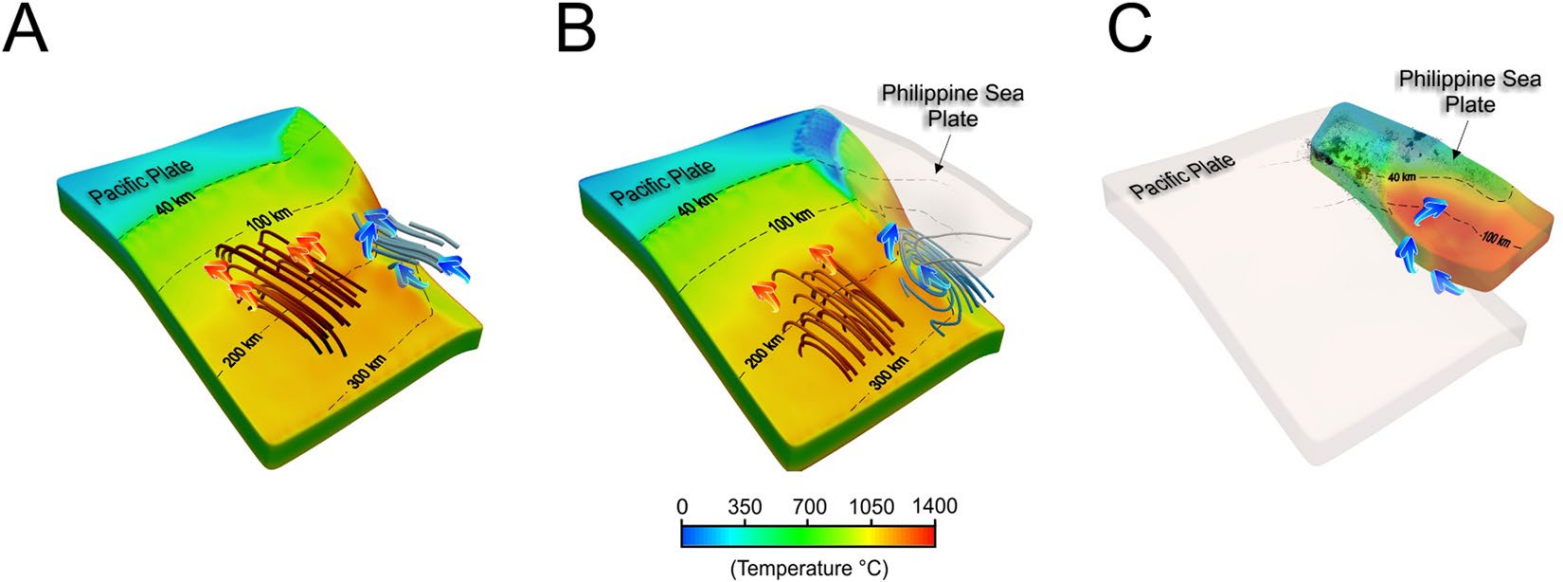
Slab surface temperature and mantle flow distribution. (**A**) Numerical experiment with a single plate (Pacific). Red and blight blue tubes depict the mantle wedge flow in two regions. Note the trench normal mantle flow in both areas (shown as red and blue arrows). (**B**) Numerical experiment with double subduction (Philippine Sea plate is shown as a semitransparent surface). Note the complex toroidal mantle flow underneath the Philippine Sea plate. (**C**) Temperature distribution inside the Philippine Sea plate. Black dots represent seismicity. Pacific plate is shown as a semitransparent surface. Blue arrows represent the mantle flow around the bottom of the Philippine Sea plate.

Another consequence of dual subduction is a complex toroidal mantle wedge flow around the bottom of the Philippine Sea slab edge, which mainly depends on the interaction between the two oceanic slabs (Fig. 4). Laboratory and numerical experiments of single-slab subduction already revealed a great complexity of mantle circulation depending on the particular settings for a subduction zone. In general, trench rollback enhances flow in the mantle wedge, and around the leading edge of the sinking slab^29,30^. The dynamics of dual slabs started to be investigated only recently^15,26,31,32^ and revealed a complex interaction pattern, depending on the relative position of both slabs. For trench parallel subduction systems, the two slabs are not fully independent but rather interact through viscous stresses induced by asthenospheric flow^31^. Another case of double subduction comprising the subduction on both sides of one single oceanic plate, the flow pattern is dominated by a lateral escape of the subslab mantle through rollback-induced flow^32^. Our double subduction model also reveals a strong mantle return flow induced by the Pacific plate subduction beneath Kanto which exceeds locally the mantle return flow induced by the Philippine Sea plate (Fig. S2). Compared with the model of single subduction, the enhanced mantle flow in the sandwiched mantle wedge beneath Kanto can reach shallower loci along the strike of the Pacific plate (Fig. 4A and B). The increased mantle return flow facilitated by the double subduction of the Pacific and the Philippine Sea plates transport increases the Philippine Sea plate temperature (Fig. 2B) and limit the maximum extent of intraslab seismicity to shallower depths of 30 km to 100 km (Fig. 4C). Considering that brittle failure to plastic deformation transition inside the slab occurs at temperature <750 °C^33–35^, we observed a good correlation with the Philippine Sea plate seismicity (Fig. S4). This suggests that temperature inside the Philippine Sea plate plays a key control on the maximum depth extent for seismic activity.

In this study, combined precise seismological observations and robust numerical modelling results show that the subduction of the Philippine Sea plate on top of the Pacific plate beneath Kanto plays is a key process that contributes to significant variations of water content and seismicity distribution, corresponding to the oceanic crust.

## Methods

### Model parameters and boundary conditions

The modelling was performed resolving the governing equations describing the conservations of mass, momentum, and energy for calculating temperature, flow velocity, and pressure, assuming an anelastic liquid approximation^14,36^. The three-dimensional numerical model simulates subduction of the oceanic Pacific and Philippine Sea plates beneath a fixed continental plate. The computations are performed within a Cartesian domain 800 km long, 700 km wide and 400 km deep (Fig. S3). This domain is evenly divided into grid cells, which corresponds to an 10 × 10 × 4 km grid resolution. Compared with our previous study^15^, in this work we carried out these numerical simulations with more than twice increase in spatial resolution.

The mechanical boundary conditions are as follows: the top boundary is rigid (Dirichlet) and the bottom boundary is permeable (Neumann) in the vertical direction; lateral boundaries are also permeable (Neumann) except the lateral boundaries corresponding to the oceanic Pacific and Philippine Sea plates, which have prescribed a time-dependent subduction velocity. We considered the two oceanic slabs in close contact with each other with no gaps along the slab contact zone. Viscous decoupling is not included on the megathrust and slab contact zone due to uncertainty. The age of the Pacific plate is considered to be fixed at 130 Myr^37^, and its current thickness is 85 km, whereas the age of the Philippine Sea plate beneath Kanto is considered to be also fixed at 40 Myr^38^ and a current slab thickness of 60 km^39,40^, calculated using a half space cooling model with the temperature at the base of lithosphere of 1100 **°**C and the mantle temperature of 1400 **°**C. Our models include a well-constrained^41^ constant convergence rates for both Pacific (9.7 cm/yr) and Philippine Sea (5.4 cm/yr) plates. However, for the Philippine Sea plate, the plate motion velocity changes abruptly its direction at 3 Ma^42^ from N26.5°W to N59.8°W^40^. The subduction evolution of the Pacific plate is integrated in time 20 Myr, whereas the subduction history of the Philippine Sea plate is considered to be only 9 Myr, which is the time required for the leading edge to reach its current location. The subduction histories timing assumed for the two plates ensured that the calculated plate geometry at 0 Ma matched the current plate geometry based on seismic tomography^8^. In terms of rheology, we use a composite upper mantle viscosity for deformation at constant stress, where model viscosity is defined by the viscous flow law for wet olivine^43,44^ (see model parameters for the diffusion and dislocation creep of olivine in Supplementary Table 1). Slab H_2_O content is calculated for P-T conditions computed from the phase diagrams^20–22^.

### Model sensitivity tests

We performed sensitivity tests to investigate the robustness of our modelling results, and varied the mantle viscosity from 0.9 × 10^20^ Pa s to 1.1 × 10^20^ Pa s, and mantle density from 3250 kg/m^3^ to 3350 kg/m^3^. We present the benchmark model results as deviation from the reference models (**Δ**T and **Δ**H_2_O), and show these results at different depth levels within the Pacific slab. The tests show that mantle density variations (±50 kg/m^3^) induce small temperature variations of ~10 **°**C at depths <100 km, and ~30 **°**C at 200 km depth (Figs S5 and S6). In terms of H_2_O content, the Pacific crust shows no significant variations. However at higher depths within the Pacific slab the differences in H_2_O estimates are up to 10%, but they are rather limited and concentrated in small regions. Mantle viscosity variations tests show maximum temperature variations of only ~10–20 **°**C and concentrated at depths of 40–100 km. Similarly with the mantle density variation tests, in terms of H_2_O content, the Pacific crust shows almost no significant variations. Only at higher depths within the slab the differences in H_2_O estimates are up to 8% but they are limited and concentrated in small regions (Figs S7 and S8).

## Electronic supplementary material

Supplementary Information
