# Supplementary material for: Seismogenesis of dual subduction beneath Kanto, central Japan controlled by fluid release
Source: Sci Rep. 2017 Dec 4;7:16864. doi: 10.1038/s41598-017-16818-z (PMC5714997; doi:10.1038/s41598-017-16818-z)
Supplement: Supplementary file 1 — Supplementary Information [file 41598_2017_16818_MOESM1_ESM.pdf]

Supplementary Information for

Seismogenesis of dual subduction beneath Kanto, central Japan controlled by fluid release

Yingfeng Ji[1], Shoichi Yoshioka[1,2], Vlad C. Manea[3,4*] and Marina Manea[3,4]

[1] *Research Center for Urban Safety and Security, Kobe University, Rokkodai-cho 1-1, Nada ward, Kobe 657-8501, Japan*

[2] *Department of Planetology, Graduate School of Science, Kobe University, Rokkodai-cho 1-1, Nada ward, Kobe 657-8501, Japan*

[3] *Computational Geodynamics Laboratory, Centro de Geociencias, Universidad Nacional Autónoma de México, Campus Juriquilla, Querétaro, 76230, Mexico*

[4] *Astronomical Institute of the Romanian Academy, 040557, Bucharest, Romania*

[*] *vlad@geociencias.unam.mx*

Contents of this file

1. Supplementary model settings
2. Supplementary Figure S1, Figure S2, Figure S3, Figure S4, Figure S5, Figure S6, Figure S7, and Figure S8.
3. Supplementary Tables 1 and 2
4. Supplementary References

Introduction

The supporting information provides the supplementary model settings, figures (S1, S2, S3, and S4), Tables 1 and 2, and References.

1. Supplementary Information

Model settings

The governing equations are the conservations of mass, momentum, and energy following the anelastic liquid approximation (Ji et al., 2016; 2017):

$$\nabla (\rho \mathbf{v}) = 0 , \tag{S1}$$

$$-\nabla P + \nabla(\nabla \boldsymbol{\tau}) - \delta_{i3}\rho g \alpha \Delta T = 0 , \text{ and} \tag{S2}$$

$$\rho C_p \left(\frac{\partial T}{\partial t} + \mathbf{v} \cdot \nabla T\right) = k\nabla^2 T + \boldsymbol{\tau}\dot{\boldsymbol{\varepsilon}} + \delta_{i3}\rho g \alpha T v_3 + \rho H_r , \tag{S3}$$

where ρ is the density, $\mathbf{v}$ is the flow velocity vector, P is the pressure, $\boldsymbol{\tau}$ is the deviatoric stress tensor, δ_{ij} is the Kronecker delta where i and j are spatial indices, g is the gravitational acceleration, α is the reference thermal expansivity, ΔT is the temperature difference relative to the surface, C_p is the specific heat at constant pressure, T is the temperature, k is the thermal conductivity, $\dot{\boldsymbol{\varepsilon}}$ is the strain rate tensor, v_3 is the flow velocity component in the vertical direction, and H_r is the internal radioactive heating per unit mass. The energy equation includes an advection term, $\rho C_p \mathbf{v} \cdot \nabla T$; a thermal diffusion term, $k\nabla^2 T$; a viscous dissipation, $\boldsymbol{\tau}\dot{\boldsymbol{\varepsilon}}$; an adiabatic heating term, $\delta_{i3}\rho g \alpha T v_3$; and a radioactive heating term, ρH_r.

We developed a 3D kinematic thermal model based on Stag3D (Tackley and Xie, 2003) to simulate the thermal regime associated with plate subduction. The finite volume method (FVM) MPDATA by Smolarkiewicz (1984) is used to advect the temperature field, while finite differences method (FDM) are used for the other terms in energy equation (Tackley and Xie, 2003). The oceanic

plate subducts into the mantle following the kinematically prescribed guide according to the slab geometry determined by seismic tomography. The thermal regime of the model evolves with time and reaches a quasi-static state after the subducted plate touches the vertical boundary on other side of the trench. We divided the modeled domain into upper crust, lower crust, slab, and mantle with respectively prescribed physical properties, e.g., including density, viscosity, and thermal conductivity (Table S2).

We establish two trenchward temperature boundary conditions for the subducting Pacific and Philippine Sea plates, and make the two incoming plates gradually subduct along prescribed guides beneath the overriding continental plate at a velocity in conform with MORVEL (DeMets et al., 2010) and NNR-MORVEL56 (Argus et al., 2011). We define the subduction velocity for the plate geometry and assuming a subduction velocity for the oceanic plate. The velocity components are obtained in a Cartesian coordinate system following the slope gradient along the subduction direction on the upper surface of the oceanic plate by hypothesizing that the slab geometry is fixed:

$$v_x(x,y,z) = \frac{-2a(x,y)b(x,y)v_y + \sqrt{\{2a(x,y)b(x,y)v_y\}^2 - 4\{a(x,y)^2+1\}[\{a(x,y)^2+1\}v_y{}^2 - v^2]}}{2\{a(x,y)^2+1\}}, \quad \text{(S4)}$$

$$v_y(x,y,z) = v_y, \text{ and} \quad \text{(S5)}$$

$$v_z(x,y,z) = a(x,y)v_x + b(x,y)v_y, \quad \text{(S6)}$$

with

$$a(x,y) = \frac{1}{2}\{Z(x+\Delta x,y) - Z(x-\Delta x,y)\}\cdot\frac{z_{\max}}{x_{\max}}, \quad \text{and} \quad \text{(S7)}$$

$$b(x,y) = \frac{1}{2}\{Z(x+\Delta x,y+\Delta y) - Z(x+\Delta x,y) + Z(x-\Delta x,y) - Z(x-\Delta x,y-\Delta y)\}\cdot\frac{z_{\max}}{y_{\max}}, \quad \text{(S8)}$$

where v is the total subduction velocity, and $v_x(x,y,z)$, $v_y(x,y,z)$, and $v_z(x,y,z)$ are the velocity components associated with the subduction of the oceanic plate in the +x, +y, and +z directions, respectively. Thus, $Z(x+\Delta x, y+\Delta y)$ is the depth at the grid $(x+\Delta x, y+\Delta y)$, and so on. Δx and Δy are the intervals of two neighboring grids along the x- and y-axes, respectively. $x_{\max}$, $y_{\max}$, and $z_{\max}$ are the dimensions of our model.

Depth distributions of the time-dependent thermal structure at the trench and trough follow the plate cooling model to predict the seafloor heat flow [McKenzie, 1967; Stein and Stein, 1992; Grose and Afonso, 2013] with a lithospheric basal temperature T_m:

$$T(z,t_{oc}) = T_m\left[\frac{z}{d_0} + \frac{2}{\pi}\sum_{n=1}^{\infty}\frac{1}{n}\sin(\frac{n\pi z}{d_0})\exp(\frac{-n^2\pi^2\kappa t_{oc}}{d_0^{\ 2}})\right], \quad \text{(S9)}$$

where $T(z,t_{oc})$ is the temperature at depth z and age t_{oc} of the oceanic plate along the Japan Trench and the Sagami Trough, d_0 is the depth below which adiabatic heating is applied, and κ is the thermal diffusivity. We calculate the thermal regime at depths beneath the trench and trough at each time step.

The interplate frictional heating and viscous decoupling are not included. This is because the observed surface heat flow is abnormally low in Kanto (Yoshioka et al., 2015) and also because megathrusts that produce great earthquakes, such as the Tohoku-Oki event, tend to be weaker and therefore dissipate less frictional heat to the surface (Gao and Wang, 2014). For the entire model, the viscous flow law for wet olivine (Burkett and Billen, 2010) following laboratory experiments is considered in this study. The deformation of olivine occurs by both diffusion creep (df) and dislocation

creep (ds), where each mechanism accommodates a portion of the total strain rate (Hirth and Kohlstedt, 2003):

$$\dot{\varepsilon}_t = \dot{\varepsilon}_{df} + \dot{\varepsilon}_{ds} . \tag{S10}$$

The composite upper mantle viscosity for deformation at constant stress is

$$\eta_{comp} = \frac{\eta_{df}\eta_{ds}}{\eta_{df} + \eta_{ds}} . \tag{S11}$$

Here, η_{df} and η_{ds} represent the diffusion creep and dislocation creep viscosities for olivine, respectively. The viscosity law is

$$\eta_{df,ds} = \left(\frac{d^p}{A_0 C_{OH}^r}\right)^{\frac{1}{n}} \dot{\varepsilon}_E^{\frac{1-n}{n}} \exp\left(\frac{E_0 + P_l V_0}{n_0 R T_a}\right), \tag{S12}$$

where $\dot{\boldsymbol{\varepsilon}}_{\mathbf{E}} = (\frac{1}{2}\dot{\varepsilon}_{ij}\dot{\varepsilon}_{ij})^{\frac{1}{2}}$ is given by the square root of the second invariant of the strain rate tensor reported by Ranalli (1995), A_0 is the preexponential factor, d is the grain size, p is the grain size exponent, C_{OH} is the OH concertration (H/10^6Si), r is the C_{OH} exponent, n and n_0 are the stress exponents, E_0 is the activiation energy, V_0 is the activiation volume, T_a is the temperature, including the adiabatic temperature gradient (3×10^{-4} K/m), R is the gas constant, and P_l is the lithostatic pressure (Turcotte and Schubert, 2002). The parameters of the composite viscosity are described in Hirth and Kohlstedt (2003). The slab geometry used is constrained by the seismic topography provided by Tokyo Institute of Technology, Japan (Nakajima, et al., 2009). The original data provided is an isodepth xyz (lon-lat-depth) file for upper surface of the subducted plate. We checked the slab geometry in Paraview (using a function for generation of isodepth contouring) and we compared the Paraview isodepth data with the original isodepth file to ensure they are as far as consistent with each other.

Petrological modelling

Ultramafic mantle rocks as harzburgite (olivine + orthopyroxene) represent the dominant rock type in mantle wedges and the uppermost oceanic mantle, and depleted lherzolite (olivine + orthopyroxene + clinopyroxene) considered as subordinate (Hacker et al., 2003). Seismological studies support the hypothesis that harzburgite represents the principal rock type in the upper mantle. The observed P wave speeds from White et al. (1992) for oceanic lower crust and mantle compared with P wave speeds for various rocks at 200 MPa (Hacker et al., 2003) indicate that most oceanic uppermost mantle (suboceanic mantle) velocity measurements are best explained in terms of spinel harzburgite mantle composition. Due to the above reasons, in our petrological modeling approach harzburgite is assumed as the dominant ultramafic rock.

We established a P-T-wt%-facies database according to Omori et al. (MORB, 2009) and Hacker et al. (2003) with a P-T grid interval of 0.04 GPa (1.2 km) and 5℃. The temperature and pressure at every P-T grid point is calculated from our 3D thermal model. The pressure (GPa) at every grid point is obtained by converting its depths (km) through PREM (Preliminary Reference Earth Model) parameters. Using the temperature and pressure provided by our simulations we estimate both each facies domain and the corresponding water content (wt%) at every grid.

2. Supplementary figures and legends

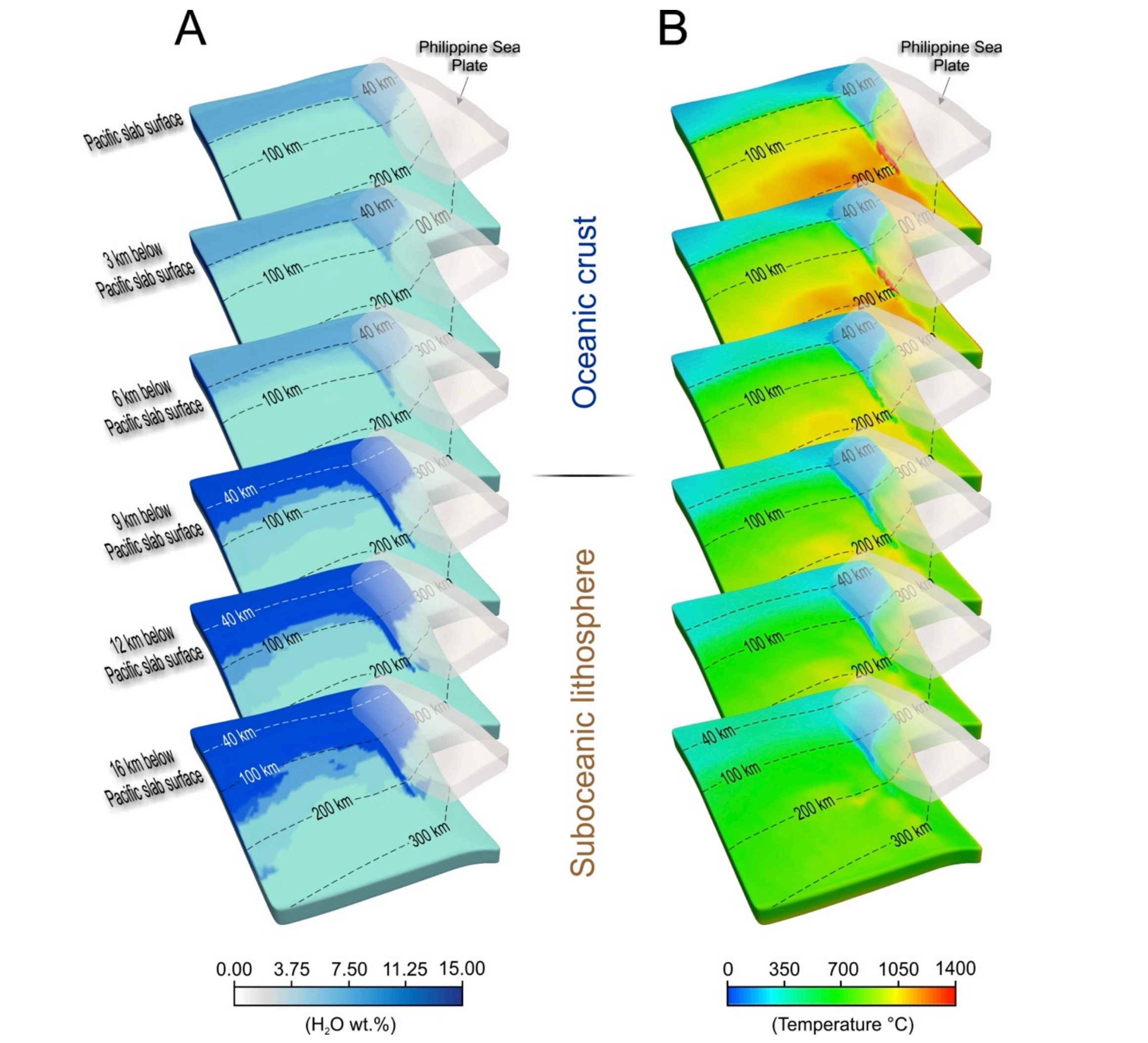

Figure S1. H_2O and temperature distribution at different depths within the Pacific plate.

A. Distribution of maximal estimates of H_2O content at different depths (surface, 3, 6, 9, 12, and 16 km) below the Pacific slab surface. **B.** Distribution of temperature at different depths (surface, 3, 6, 9, 12, and 16 km) below the Pacific slab surface. Note how the temperature increase due to the contact with the Philippine Sea plate is confined within the first half of the Pacific oceanic crust, whereas the cooling effect conducts deeper inside the plate.

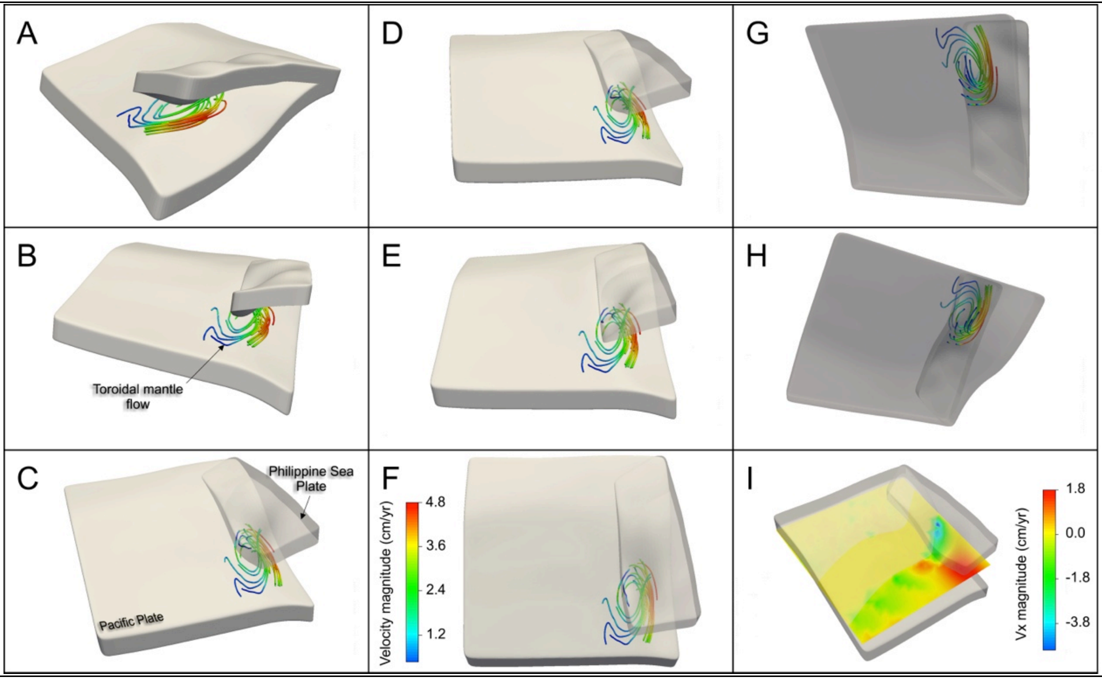

Figure S2. Mantle flow distribution and horizontal velocity through the gap between the Philippine Sea plate and the Pacific plate. Philippine Sea and Pacific slab are shown as solid volumes (a,b) and semitransparent volume for the Philippine Sea plate (c,d,e,f). g),h) View from below the Pacific plate (shown as semitransparent volume). Streamlines are shown as colored thick tubes, and the colors represent mantle flow velocities. i) Horizontal velocities along a 2D surface cut through the gap between the Philippine Sea and Pacific slabs.

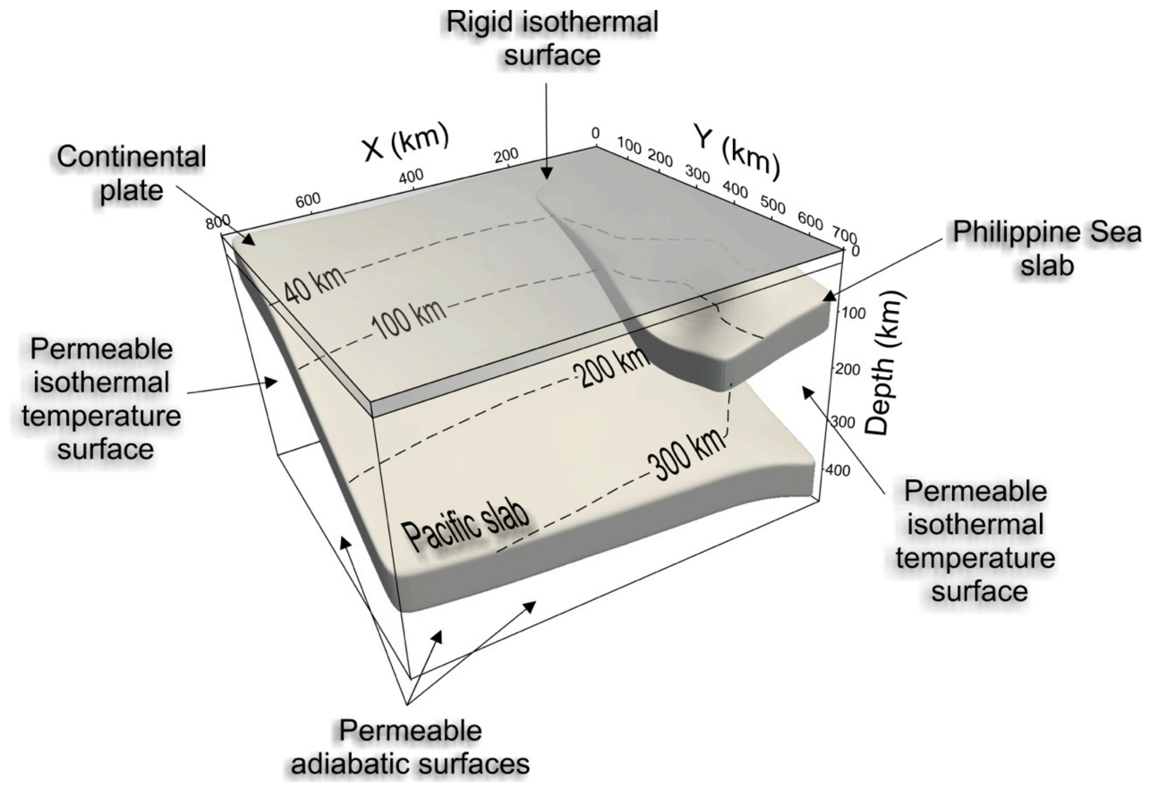

Figure S3. Model setup. The computations are performed within a Cartesian domain (800 km long, 700 km wide and 400 km deep) evenly divided into grid cells, which corresponds to an 10 × 10 × 4 km grid resolution.

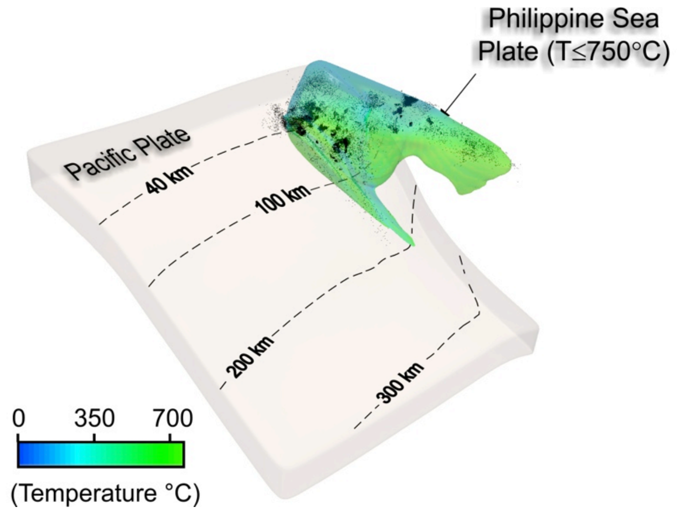

Figure S4. Philippine Sea plate seismicity and slab temperature. Philippine Sea slab is shown as 750°C isothermal semitransparent volume. Black dots represent seismicity.

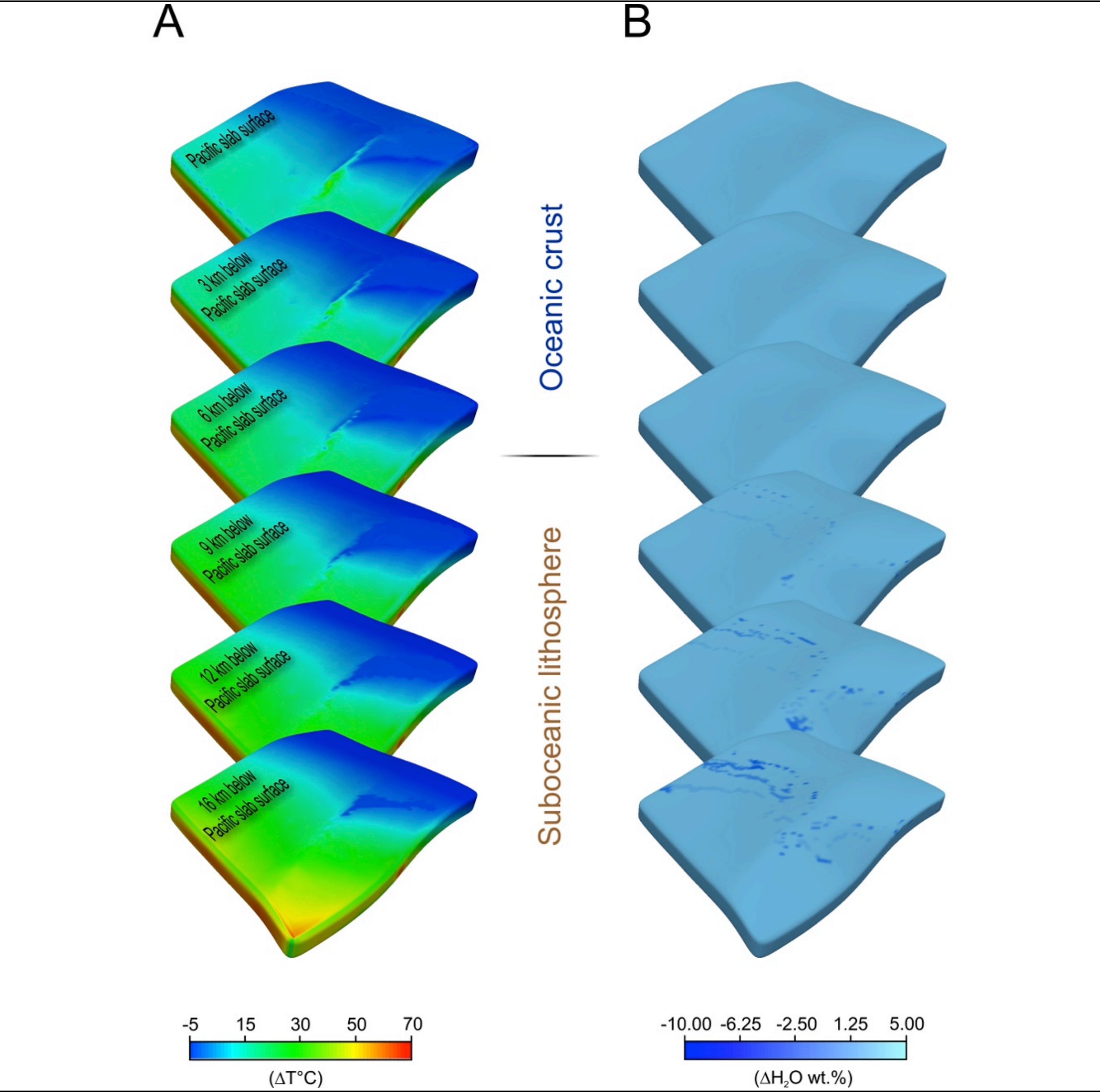

Figure S5. Temperature and H_2O variations for mantle density variation of -50 kg/m^3 (mantle density of 3250 kg/m^3).

A. Distribution of temperature variation at different depths (surface, 3, 6, 9, 12, and 16 km) below the Pacific slab surface. **B.** Distribution of maximal estimates variations of H_2O content at different depths (surface, 3, 6, 9, 12, and 16 km) below the Pacific slab surface.

137

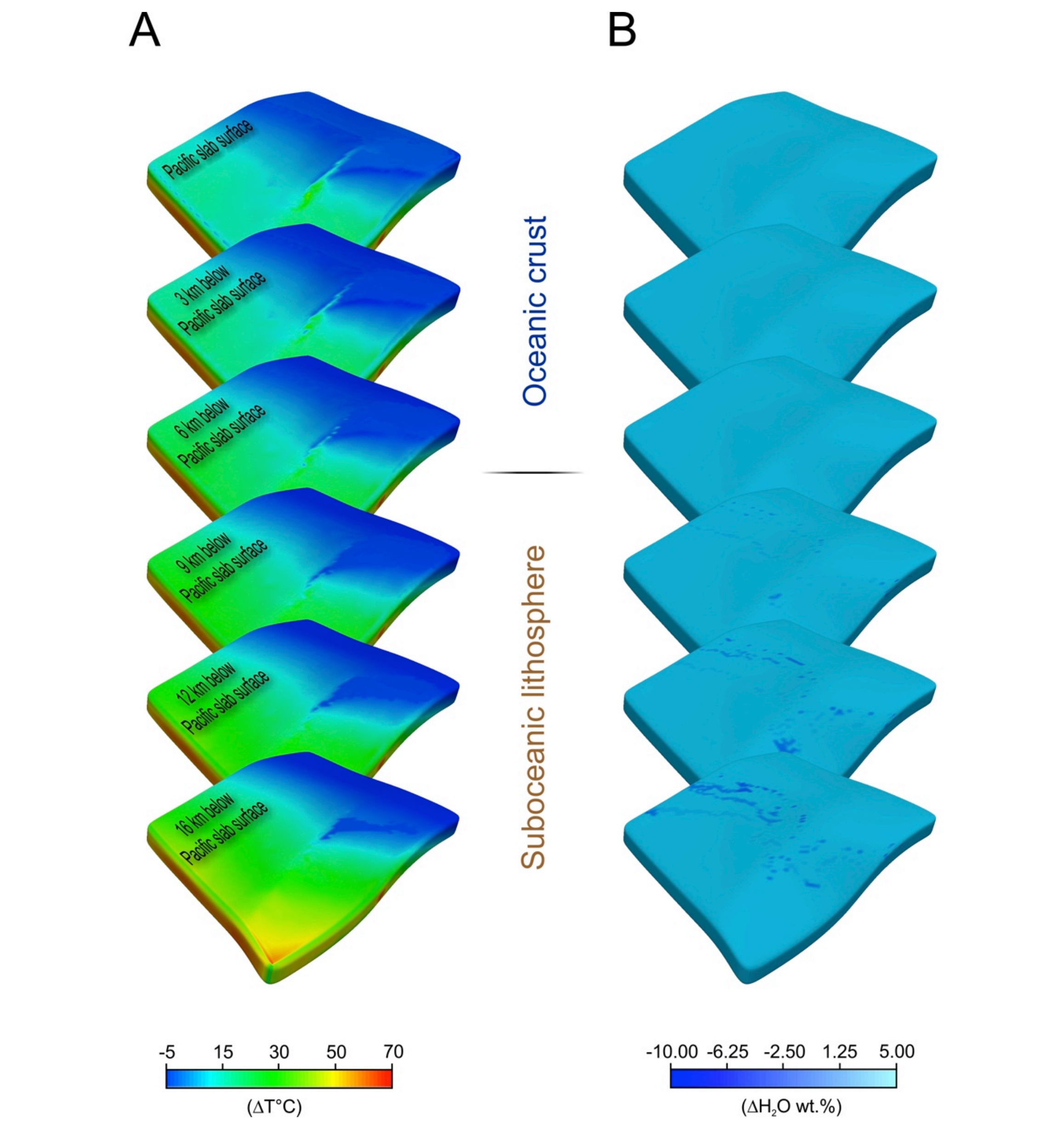

Figure S6. Temperature and H_2O variations for mantle density variation of +50 kg/m^3 (mantle density of 3350 kg/m^3).

A. Distribution of temperature variation at different depths (surface, 3, 6, 9, 12, and 16 km) below the Pacific slab surface. **B.** Distribution of maximal estimates variations of H_2O content at different depths (surface, 3, 6, 9, 12, and 16 km) below the Pacific slab surface.

138

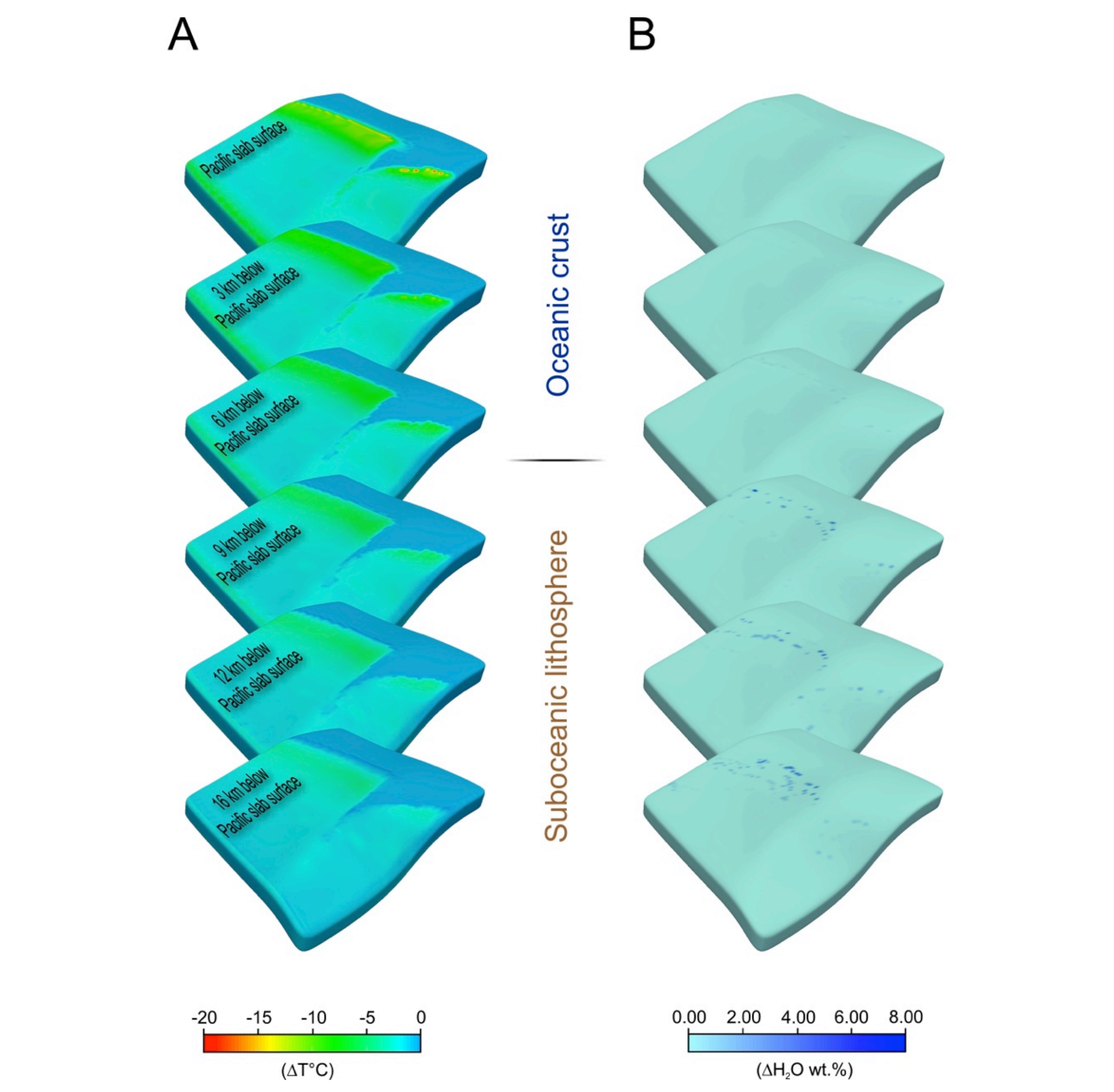

Figure S7. Temperature and H_2O variations for mantle viscosity variation of -10^{-1} Pa s (mantle viscosity of 0.9×10^{20} Pa s).

A. Distribution of temperature variation at different depths (surface, 3, 6, 9, 12, and 16 km) below the Pacific slab surface. **B.** Distribution of maximal estimates variations of H_2O content at different depths (surface, 3, 6, 9, 12, and 16 km) below the Pacific slab surface.

139

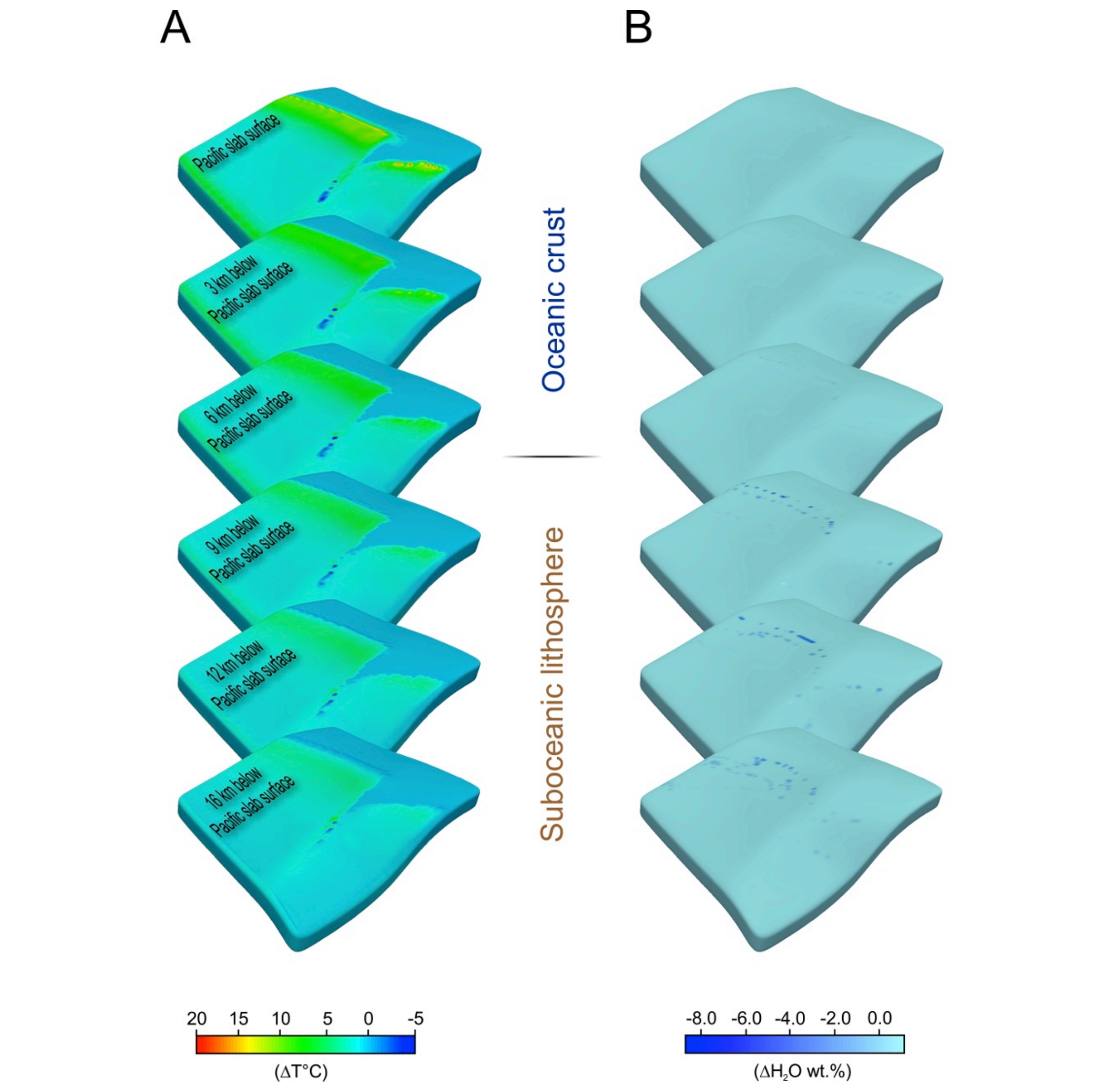

Figure S8. Temperature and H_2O variations for mantle viscosity variation of $+10^{-1}$ Pa s (mantle viscosity of $1.1x10^{20}$ Pa s).

A. Distribution of temperature variation at different depths (surface, 3, 6, 9, 12, and 16 km) below the Pacific slab surface. **B.** Distribution of maximal estimates variations of H_2O content at different depths (surface, 3, 6, 9, 12, and 16 km) below the Pacific slab surface.

3. Supplementary Table 1. Model parameters for the diffusion and dislocation creep of olivine (Armienti and Tarquini, 2002; Hirth and Kohlstedt, 2003; Burkett and Billen, 2010)

	**Flow law parameters**		
	Parameter	Diffusion creep	Dislocation creep
n_0	Stress exponent	1.0	3.5
A_0	Preexponential factor ($s^{-n}Pa^{-n}\mu m^{p}H^{-r}\ 10^{6r}Si^{r}$)	1.0	9.0×10^{-20}
E_0	Activiation energy (kJ/mol)	335	480
V_0	Activiation volume (m^3/mol)		
	Upper mantle	$4.0\times10^{--6}$	$11.0\times10^{--6}$
	Lower mantle	$1.5\times10^{--6}$	-
d	Grain size (μm)		
	Upper mantle	10,000	-

	Lower mantle	40,000	-
p	Grain size exponent	3.0	-
C_{OH}	OH concertration ($H/10^6Si$)	1000	1000
r	C_{OH} exponent	1.0	1.2

Table 2. Model parameters for each domain

Domains	Upper crust	Lower crust	Slab	Mantle	Accretionary prism
Density (kg/m^3)	2600	2900	3300	3300	2600
Viscosity (Pa·s)	1×10^{20}	1×10^{20}	1×10^{20}	1×10^{20}	1×10^{20}
Thermal conductivity (W/m·K)	2.5	2.5	2.5	2.5	1.4
Radioactive heat (W/m^3)	$7.3e^{-10}$	$1.4e^{-10}$	2.245^{e-13}	2.245^{e-13}	$7.3e^{-10}$

4. Supplementary References

Argus, D. F., Gordon R. G. and C. DeMets (2011). Geologically current motion of 56 plates relative to the no-net-rotation reference frame, *Geochem. Geophys. Geosyst.*, 12, Q11001, doi:10.1029/2011GC003751.

Armienti, P., Tarquini, S. (2002). Power law olivine crystal size distributions in lithospheric mantle xenoliths. *Lithos*, 65(3), 273-285.

Bassett, D., R. Sutherland, and S. Henrys (2014) Slow wave speeds and fluid overpressure in a region of shallow geodetic locking and slow slip, Hikurangi subduction margin, New Zealand, *Earth Planet. Sci. Lett.*, 389, 1–13, doi:10.1016/j.epsl.2013.12.021.

Burkett, E. R. and M. I. Billen (2010) Three-dimensionality of slab detachment due to ridge-trench collision: laterally simultaneous boudinage versus tear propagation. *Geochem. Geophys. Geosyst.*, 11, Q11012, doi:10.1029/2010GC003286.

DeMets, C., Gordon, R. G. and D. F. Argus (2010). Geologically current plate motions, *Geophys. J. Int.*, 181, 1, 1-80, doi: 10.1111/j.1365-246X.2009.04491.x.

Gao, X., Wang, K. (2014). Strength of stick-slip and creeping subduction megathrusts from heat flow observations. *Science*, 345(6200), 1038-1041.

Grose, C.J., Afonso, J.C. (2013), Comprehensive plate models for the thermal evolution of oceanic lithosphere, *Geochem. Geophys. Geosyst.*, 14, 3751–3778, doi:10.1002/ggge.20232.

Hacker, B.R., Abers, G.A., Peacock, S.M. (2003). Subduction factory 1. Theoretical mineralogy, densities, seismic wave speeds, and H2O contents. *J. Geophys. Res.*, 108, 2029, doi:10.1029/2001JB001127.

Hirth, G. and D. Kohlstedt (2003) Rheology of the upper mantle and the mantle wedge: A view from the experimentalists, Inside the Subduction Factory, *Geophys. Monogr. Ser.*, vol. 138, edited by J. Eiler, pp. 83–105, AGU, Washington, D. C.

Ji, Y., S. Yoshioka, and T. Matsumoto (2016) Three-dimensional numerical modeling of temperature and mantle flow fields associated with subduction of the Philippine Sea plate, southwest Japan, *J. Geophys. Res.*, 121, 4458–4482, doi:10.1002/2016JB012912.

Ji, Y., S. Yoshioka, V. C. Manea, M. Manea, and T. Matsumoto (2017), Three-dimensional numerical modeling of thermal regime and slab dehydration beneath Kanto and Tohoku, Japan, *J. Geophys. Res. Solid Earth*, 122, 332–353, doi:10.1002/2016JB013230.

McKenzie, D.P. (1967). Some remarks on heat flow and gravity anomalies, *J. Geophys. Res.*, 72, 6261–6273.

Nakajima, J., Hirose F., Hasegawa A. (2009). Seismotectonics beneath the Tokyo metropolitan area, Japan: Effect of slab-slab contact and overlap on seismicity, *J. Geophys. Res.*, 114, B08309, doi:10.1029/2008JB006101.

Omori, S., Kita, S., Maruyama, S., Santosh, M., (2009). Pressure–temperature conditions of ongoing regional metamorphism beneath the Japanese Islands, *Gondwana Research*, 16, 458−469.

Ranalli, G. (1995) *Rheology of the Earth*, 76 pp., 2nd ed., Chapman and Hall, London.

Stein, C.A., and S. Stein (1992), A model for the global variation in oceanic depth and heat flow with lithospheric age, *Nature*, 359, 123–129.

Tackley, P.J., Xie, S. (2003). Stag3D: A code for modeling thermo-chemical multiphase convection in Earth's mantle, Computational Fluid and Solid Mechanics 2003, edited by K. J. Bathe, pp. 1524−1527, Elsevier B.V., Amsterdam, Netherlands.

Turcotte, D. L., Schubert, G. (2002) *Geodynamics*, 2nd ed., 186 pp., Cambridge Univ. Press, Cambridge, U. K.

White, R.S., McKenzie, D., O'Nions R.K. (1992). Oceanic crustal thickness from seismic measurements and rare earth element inversions, *J. Geophys. Res.*, 97, 19683−19715.

Yoshioka, S., Takagi, R., Matsumoto, T. (2015). Relationship between temperatures and fault slips on the upper surface of the subducting Philippine Sea plate beneath the Kanto district, central Japan. *Geophys. J. Inter.*, 201(2), 878-890.
